# Food Waste along the Food Chain in Romania: An Impact Analysis

**DOI:** 10.3390/foods10102280

**Published:** 2021-09-26

**Authors:** Oana M. Dumitru, Corneliu S. Iorga, Gabriel Mustatea

**Affiliations:** National Research & Development Institute for Food Bioresources, 020323 Bucharest, Romania; oanamniculae@yahoo.com (O.M.D.); gabi.mustatea@bioresurse.ro (G.M.)

**Keywords:** food waste, food chain impact, food waste mitigation, food waste drivers

## Abstract

Food waste is a hot topic around the world due to the significant environmental challenge it poses. The study aims to assess the impact of food waste on the food chain at the national level. The data were obtained from quantitative impact studies, carried out in a project funded by the Ministry of Agriculture and Sustainable Development, “Methods to reduce food waste on the agri-food chain, at national level, to prevent and reduce socio-economic impact, until 2030”. A total of 852 companies were interviewed, with a turnover of almost 6.5 billion euro and a number of over 69 thousand employees, including 273 primary production enterprises, 270 food processing units, 171 distribution/retail units, and 138 HoReCa units.

## 1. Introduction

The “Farm to Fork Strategy” of the European Commission aims to make food systems fair, healthy, and environmentally friendly. The end-to-end process of “Farm to Fork” is represented by the food life cycle (handling, storage, processing, distribution, and consumption). Food loss and food waste are generated at every step of this life cycle [1,2,3].

The amount of produced food wasted among the food chain is quite high, reaching up to 50% [4,5,6].

The food waste (FW) topic has started to attract the attention of governments, non-governmental organizations (NGOs), and other actors involved in the food supply chain based on several factors, such as environmental impacts associated with the inefficient use of natural resources (water, energy, and land) and disposal to landfill, which cause pollution [4,6,7,8,9,10], morality issues (a large amount of food is wasted while millions of people around the world suffer from hunger) [4,11], and economic impact [12]. Despite the mentioned growing attention, FW is still affected by a lack of a consensus regarding definition, scope, causes, and even reporting methods. The costs associated with FW are often undervalued and underreported. Several studies reported the fact that food is predominantly thrown away, especially in developed countries, at the consumption stage of the supply chain, while food waste from residents is higher than that of businesses [13,14,15,16,17,18].

Despite the fact that research on FW has grown consistently during the last 10 years [16], calls for further studies are still needed [19], especially at the household–consumer level [17]. Furthermore, the role of the consumers in preventing FW is crucial [20,21].

A study published in 2016 by the Swedish Environmental Research Institute [22] indicates a value of over 88 million tonnes of food waste generated annually in the EU. The costs associated to this amount are estimated at 143 billion euros [23,24]. At a global scale, it is estimated that a total of 1.3 billion tons per year is wasted, of which 413 Mt is wasted in the agricultural production stage; 293 Mt in the post-harvest, handling, and storage phases; 148 Mt in the processing stage; 161 Mt in the distribution stage; and 280 Mt through household consumption [25,26].

It is essential to reduce FW by acting on the entire food chain, as generally considered also in several previous studies [27,28,29,30,31,32,33].

**Figure 1:**
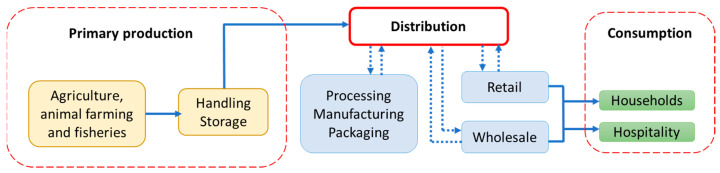
FW occurrence in different stages of food supply chain.

When we talk about the food supply chain, we must consider it as a combination of interactions between farmers and consumers connected with different food processing and distribution companies [34,35,36,37]. Factors affecting FW are, as mentioned above, numerous. In Figure 1, a schematic view of all the stages in the food supply chain where FW may occur is presented [37].

The aim of this study is to assess the impact of food waste (FW) at the national level. It covers the upper chains of the food chain, from primary production to HoReCa services. The data were obtained from impact studies conducted within a national project entitled “Methods for reducing food waste in the agri-food chain, at national level, in order to prevent and reduce the socio-economic impact, by 2030”.

## 2. Materials and Methods

Data collection for the four links of the food chain (public catering—HoReCa, distribution, processing, and primary production) was carried out between June and September 2020. A total of 852 questionnaires were applied to the representatives of the economic operators in each link in the food chain as follows: 273—primary production (NACE code 01xx, 03xx), 270—processing (NACE code 10xx), 171—distribution (NACE 46xx, 47xx), and 138—public catering—HoReCa (NACE 56xx). For the application of the 852 questionnaires, 10,987 calls were generated, out of which 6615 were returned. The time interval of the calls was 10 a.m.–5 p.m. A total of 4372 companies were contacted: total applied—852; total refusals—1392; total “I do not answer”—1980; total non-existent—130; total ineligible—18. The methods used for data collection were CATI (Computer-Assisted Telephone Interviewing) and CAWI (Computer-Assisted Web Interviewing)—exclusively in the case of respondents who expressly requested this method. Databases with Romanian companies were purchased in order to conduct the study. The first 800 economic agents in Romania were selected from each link of the food chain, according to the turnover registered in 2018. Contacting the respondents and administering the questionnaire were done through the authorized call-center software system.

The characteristics of the companies involved in the study are presented in Table 1.

The questionnaires used in the study were divided into three main parts: I. information about organizations; II. perceptions and motivations related to FW; and III. awareness of public strategies to reduce food waste.

The data were processed with the SPSS Statistic Data Editor Program and Microsoft Office Excel program. Results were presented by descriptive statistics, and one-way ANOVA and bivariate methods were applied to identify and discuss correlations. The margin error of the sampling varied from 3.1% to 7.6%, for a 95% confidence level (Table 2).

## 3. Results

### 3.1. The Structure of the Target Groups

For the primary production sector, most of the responding entities are acting in cereal cropping (53%), milk and meat production represents 10%, and fruit and vegetable production represents 3%. An important share (34%) is represented by entities having other mixed activities.

Processing units’ sample is represented by the bakery sector (34%), meat processing (24%), milk and dairy (23%), canned fruits and vegetables (3%), and oil and related products (2%). The difference is represented by sectors such as ice-cream, sweets, honey, etc.

Related to distribution/retail sector, the most of the activities are centered on meat and meat products (24%), canned fruits and vegetables (11%), sweets (11%), bakery products (9%), oils (9%), dairy products (9%), and drinks (8%). Other products (19%) are represented by fresh fruits, cereals, sugar, etc.

HoReCa sector is mainly represented by restaurants (71%). Fast-food units (11%), catering units (9%), and other (9%), such as bistros, etc., were also involved in the study.

### 3.2. Attitude towards FW

#### 3.2.1. Level of Concern

The FW phenomenon is widely accepted as important. Over 60% of respondents are very interested in this issue. However, in the primary production sector (agriculture), the level of high interest is significantly lower (Table 3).

#### 3.2.2. Sources of Information

Mass media and official sources are the most trusted sources. Profile magazines are consulted mainly by professionals from agriculture but very rare by the HoReCa specialists (Table 4). The data indicates a large proportion of lack of information, up to 10% of the respondents.

### 3.3. Perception on Level of FW

The research addressed the level of FW as volumes level, as well as level compared to the total production value. A distinction was made for technological losses. Median values for FW and food loss are between 0.86%, for the food distribution sector, to 8.63%, for the HoReCa sector (Table 5).

### 3.4. Time Evolution of FW

An important aspect of the analysis also aimed to determine the perceptions of respondents of the evolution of the FW phenomenon since 2016.

The results show that the general perception indicates a rather decreasing tendency of the phenomenon. The clearest appreciation in this respect is registered among household consumers. A discordant note is found in HoReCa, where 36% of respondents perceive an increase in losses over the last 3 years (Table 6).

### 3.5. Perception on Level of FW for Different Types of Products

For each link of the food chain, products mentioned in the questionnaires were selected based on the analysis previously performed of the organization’s database.

#### 3.5.1. Primary Production

Primary production has significant losses in cereals and livestock, probably including slaughter. The vegetable and fruit sector seems to have a better management of the products, registering only 7% losses. Minimal losses are also recorded in rapeseed cultivation (Table 7).

#### 3.5.2. Processing

Food processing has higher losses in the bakery and meat industry. Losses in pastry and confectionery, as well as technological losses in meat processing, are also significant (Table 8).

#### 3.5.3. Distribution/Retail

The main sources of losses in retail are related to damaged and expired products. However, the impact of losses is rather small, with over 40% of respondents not having or considering that they do not have a waste situation in their units (Table 9).

#### 3.5.4. HoReCa

The types of products subject to losses are various food scraps (from the preparation process, unconsumed leftovers from served portions, or expired food) as well as expired raw materials. Only 3% of respondents could not or did not consider the phenomenon significant for their units (Table 10).

#### 3.5.5. Approach to the FW Level on Food Chain Scale

The analysis at the level of the entire food chain was made starting from the initially produced volume (was considered 100%) by successively applying the losses on each link from the primary agricultural production to the domestic consumers (6.5%, as determined by Dumitru et al., 2021 [38]). The calculation was made on two variants:

1. Maximized variant: the reductive hypothesis was used, according to which the weighting coefficients of all the links in the food chain are equal to 1, respectively, that the impact of each sectoral level of waste is fully reflected in the consolidated value per chain. In this variant, the HoReCa sector was integrated as an intermediary between the distribution/retail link and household consumers.
2. Reduced variant: we started from the hypothesis that the impact of the HoReCa sector in the total volume of food is insignificant, representing less than 3% of the volume of food purchased by household consumers [39], so that the impact of waste on this sector was eliminated from the calculation of the consolidated value of food waste on the whole food chain.

The results obtained define an interval in which the food waste generated throughout the chain falls (Table 11).

### 3.6. Actions to Reduce FW

Two main directions were envisaged, consisting of considerations of the best FW control measures and implementation of FW measures on local or national scales. Multiple-choice questions were used, having “Other” as an alternative for personal input. The high number of “None/Not the case and N/A” responses indicate a reduced interest of the participants in this topic.

#### 3.6.1. Most Efficient Measures Considered Useful by Entrepreneurs to Reduce FW

The options of entrepreneurs in productive links, including public catering, give as the main measure to reduce the waste of investments in new technologies, with increased efficiency. The distribution sector believes that better business management is the solution for its representatives (Table 12).

#### 3.6.2. Implemented or In-Implementation FW Control Measures along the Food Chain

The analysis of the responses received leads to a first observation related to the low level of effective involvement in the implementation of measures to reduce waste in all sectors; well over half of respondents do not know, do not apply, or do not respond. Agricultural producers reuse waste mainly for composting/fertilizer. Processors are especially looking for internal solutions to enhance some byproducts. Distributors are inclined to resort mainly to donations but also in very small proportions (4%). Finally, the food sector is tempted to optimize its supply and launch new products to encourage consumption (Table 13).

#### 3.6.3. Knowledge of Existing Measures for Reducing FW on National Scale

The analysis reveals an overwhelming proportion of ignorance by entrepreneurs in the food chain of initiatives to regulate food waste in Romania. The proportions are over 90% for the negative response groups, either denial of any measure or ignorance (Table 14).

## 4. Discussion

The FW phenomenon is widely accepted as important. It arouses great and very high interest in more than 60% of cases for all links in the food chain. The interest is at a maximum in public catering (82%).

The sources of information that interested entrepreneurs use in keeping up to date with regulations and initiatives in the field of FW control reveal the media and official sources as the main resources. Specialty magazines have a significant impact among agricultural producers and much less among catering entrepreneurs. These communication channels have and will have a critical role in the implementation of control programs and the reduction of FW.

The assessment of the impact of FW was made both at the level of each link of the food chain but also on the whole chain. The results indicate a placement close to the European average of 20% and a general level of waste in a range of 14.56% to 21.94%. In the UK, the level of FW is reported to be 22.32% [39].

The main waste-generating sectors are public catering (8.63% at the sectoral level) and household consumption (6.50% at the sectoral level). Regarding the weights in the general waste, household consumers represent up to 40.78% of the total FW in Romania. Regarding the analysis of the types of products with high waste risk, the primary production registers significant losses in the cereal field and in the zootechnical field, probably together with slaughter.

Food processing has higher losses in the bakery and meat industry. Losses in confectionery, as well as technological losses in meat processing, are also significant. The main sources of losses in retail are related to damaged and expired products. In public catering, the types of products subject to losses are represented by various food scraps (from the preparation process, unconsumed scraps from served portions or expired food) as well as expired raw materials. At the level of household consumers, the data indicate bakery products and home-cooked food as the products with the highest risk of waste.

Primary agricultural production has as its main weaknesses generating losses, outdated technologies, and the generation through primary processing of byproducts for which they do not have capitalization solutions, such as inefficient marketing. The food industry is facing problems related to the capitalization of byproducts resulting from technological processes but also problems of excessive supply of raw materials or the emergence of substandard products.

The distribution has as its critical causes generating losses, the mistakes of handling the products, and over-supply with certain assortments, which are not sold fast enough. The analysis reveals as the main cause of waste in the public alimentation the improper dimensioning of the portions offered to the clients, who do not end up consuming all the food offered. Other significant causes are leftovers resulting from menu preparation, supply malfunctions, or excess prepared food, which must be discarded at the end of the day. The analysis of consumer behavior suggests as the most common causes the incorrect scheduling of food consumption, as well as the habit of not leaving the remaining food overnight.

The study reveals major deficiencies in public communication related to the measures and regulations adopted. Over 90% of entrepreneurs are unaware of national FW control activities and initiatives.

## 5. Conclusions and Recommendations

This is the first systematic study at a national level conducted on a representative target group covering the entire food chain.

It is becoming clearer that avoidance and reduction of FW should be prioritized in order to improve food security and minimize burdens, both environmental and economic. To develop strategies for avoiding and reducing FW, is critical to have information on the scale of FW generation, its sources and causes, and associated environmental burdens.

The assessment of the impact of FW results were close to the European average of 20% (21.94%).

With regard to their own assessments of necessary measures at company level, the choices of entrepreneurs in productive links, including public catering, give as the main measure to reduce the waste of investments in new technologies, with increased efficiency. The distribution sector believes that better business management is the solution for its representatives. However, the appetite of entrepreneurs in this direction is low. A significant problem is that entrepreneurs do not correlate the chosen lines of action with the various causes, identified by them themselves, retaining conventional, often formal solutions. Agricultural producers reuse waste mainly for composting/fertilizer, processors are mainly looking for domestic solutions for the recovery of by-products, distributors are inclined to mainly access donations, but also in very small proportions, and the food sector is tempted to optimize supply and launch new products to encourage consumption. The situation also seems to be perpetuated in the short-term action plans at the level of the whole food chain.

Based on the conclusions of the study, there are several recommendations, such as intensifying public communication related to food waste using those media relevant target groups; strengthening the role that sustainable economy, reducing losses, will play in future funding programs; opening refurbishment and digitization programs at the level of all productive links of the food chain; launching programs to implement a management system at the company of sustainable principles, with detailed needs analysis and correlation lines of action need to meet those needs.

The high number of “None/Not the case and N/A” responses on FW reduction actions represents a risk of FW future evolution. Therefore, more intensive awareness actions and campaigns are recommended.

## Figures and Tables

**Table 1:** Characteristics of the companies participating to the impact assessment.

Values 2018	Number of Units	Minimum Value	Maximum Value	Total	Mean
All food chain values
Turnover (euro)	852	708,141.83	149,947,886.97	6,467,072,647	7,590,460.94
Employees	852	0	2867	69,260	81
Primary production values
Turnover (euro)	273	1,387,232	149,947,887	1,898,675,235	6,954,854
Employees	273	0	1034	17,782	65
Processing units’ values
Turnover (euro)	270	877,327	142,796,366	2,267,552,109	8,398,341
Employees	270	0	2867	34,701	129
Distribution/retail units’ values
Turnover (euro)	171	2,558,132	83,210,710	2,013,824,892	11,776,754
Employees	171	0	430	8955	52
HoReCa values
Turnover (euro)	138	708,142	24,413,962	287,020,411	2,079,858
Employees	138	0	607	7822	57

**Table 2:** Sample statistical data, along the food chain.

	Primary Production	Processing Units	Distribution	HoReCa
Questionnaires	273	270	171	138
Type	Non-probabilistic, opportunistic selection among the top 800 companies in the country by turnover in 2018, for each category			
Representativity	Margin error ± 4.8%, 95% confidence level	Margin error ± 4.9%, 95% confidence level	Margin error ± 6.5%, 95% confidence level	Margin error ± 7.6%, 95% confidence level
Hole chain representativity	Margin error ± 3.3%, 95% confidence level			
Method	CATI (Computer-Assisted Telephone Interviewing) and CAWI (Computer-Assisted Web Interviewing)			
Period	June–September 2020			

**Table 3:** Awareness level of FW impact along the food chain.

	How Concerned Are You about FW Impact on Your Business?			
	Primary production	Processing	Distribution	HoReCa
Highly concerned	36%	48%	42%	54%
Concerned	32%	26%	28%	28%
Relatively concerned	8%	9%	5%	7%
Not too concerned/Not at all	4%	4%	8%	4%
N/A	19%	13%	18%	7%

**Table 4:** Main sources of information among food chain professionals.

	Primary Production	Processing	Distribution	HoReCa
I do not inform myself	10%	5%	13%	9%
Official sources	28%	38%	30%	33%
Mass media	59%	57%	54%	60%
Profile magazines	28%	24%	19%	9%
Others	5%	3%	5%	18%
N/A	16%	10%	13%	4%

**Table 5:** Food waste levels along the food chain.

	No. of Respondents	Minimum Level (%)	Maximum Level (%)	Median Level (%)	Std. Deviation
Primary agricultural production					
Share of FW in yearly volume of production	243	0	60	4.20	10.112
Food processing					
Share of FW in yearly volume of production	243	0	60	3.79	7.993
Food distribution					
Share of FW in yearly volume of production	130	0	10	0.86	1.543
HoReCa					
Share of FW in yearly volume of production	126	0	50	8.63	9.285
Whole food chain					
Share of technological loss in yearly volume of production	681	1	9	2.64	2.493
Share of FW in yearly value of production	852	1	9	2.87	2.752

**Table 6:** Food waste evolution in time.

Food Chain Sector	Perception	Percent (%)
Primary agricultural production	FW increased	12
	FW maintained	49
	FW decreased	20
	N/A	19
Food processing	FW increased	17
	FW maintained	35
	FW decreased	35
	N/A	13
Food distribution	FW increased	19
	FW maintained	36
	FW decreased	25
	N/A	20
HoReCa	FW increased	36
	FW maintained	35
	FW decreased	26
	N/A	4

**Table 7:** Food waste structure in the primary agricultural production sector.

Product Mentioned	Percent (%)
Animal products (e.g., carcasses, heads, meat)	22
Fruits and vegetables	7
Dairy products	2
Cereals	34
Maize	15
Bakery	2
Eggs	5
Sunflower	9
Rape	3
Others	1

**Table 8:** Food waste structure in the food processing sector.

Product Mentioned	Percent (%)
Pastry	3
Bakery products	6
Confectionery	5
Meat/Sausages	7
Byproduct processing	1
Leftover fruits/vegetables/greens	3
Animal remains	5
Dairy products/Cheeses/Eggs	4
Wheat/Corn/Rice/Seeds/Sugar	3
Others	3

**Table 9:** Food waste structure in the food distribution sector.

Product Mentioned	Percent (%)
Damaged products	44
Expired products	33
Others	8
Not the case	15
N/A	25

**Table 10:** Food waste structure in the HoReCa sector.

Product Mentioned	Percent (%)
Food leftovers	25
Expired raw materials	25
Expired prepared food	26
Portions not fully consumed by the customer	59
Others	6
N/A	3

**Table 11:** Level of FW on the entire food chain.

Food Chain Sector	Median Value
Primary agricultural production sector	4.20%
Food processing sector	3.79%
Food distribution sector	0.86%
HoReCa sector	8.63%
Consumers from urban area	6.5%
**Food chain maximal level of FW—21.94%** Of which: - Input from primary agricultural production sector—4.2% - Input from food processing sector—3.63% - Input from food distribution sector—0.79% - Input from HoReCa sector—7.89% - Input from urban household’s consumers—5.43%	
**Food chain minimal level of FW—14.56%** Of which: - Input from primary agricultural production sector—4.2% - Input from food processing sector—3.79% - Input from food distribution sector—0.79% - Input from urban household’s consumers—5.94%	

**Table 12:** Considerations on best FW control measures among the food chain professionals.

Measure	Mentions Percent (%)			
	Primary Agricultural Sector	Food Processing	Food Distribution	HoReCa
Use of fertilizer waste	10	1	0	0
Donations	1	2	4	1
Valorization of byproducts internally or by marketing (e.g., incorporation into other products, animal feed)	4	11	3	1
Marketing of products at a reduced price	0	0	2	0
Selective collection	0	0	1	1
The waste to be taken over by a neutralization company	1	1	1	0
Production optimization through new technologies or supply management	0	1	2	17
Other	0	0	0	1
None/Not the case	34	63	67	2
N/A	49	19	20	67

**Table 13:** Implemented FW control measures along the food chain.

Measure	Mentions Percent (%)			
	Primary Agricultural Sector	Food Processing	Food Distribution	HoReCa
Use of fertilizer waste	1	0	0	0
Donations	0	1	3	1
Valorization of byproducts internally or by marketing (e.g., incorporation into other products, animal feed)	0	1	1	1
Marketing of products at a reduced price	0	0	2	0
Selective collection	0	0	1	1
The waste to be taken over by a neutralization company	0	0	0	0
Production optimization through new technologies or supply management	2	9	8	18
Other	1	2	1	1
None/Not the case	64	70	64	67
N/A	31	16	19	9

**Table 14:** Knowledge of existing FW control measures among food chain professionals.

Measure	Mentions Percent (%)			
	Primary Agricultural Sector	Food Processing	Food Distribution	HoReCa
Encouraging donations/ Creating food banks	1	3	4	3
Awareness campaigns	0	0	0	0
Implementing coherent supply system	0	1	1	2
Monitoring FW collection/recycling	0	1	0	1
Promoting advanced technologies	1	0	0	0
Promoting production fit to demands	0	0	0	1
Sales campaigns	0	1	2	0
Legislative measures	1	0	0	0
Other	1	2	2	0
None	71	69	76	80
N/A	24	20	15	10

## Data Availability

Data Availability Statement: The datasets generated for this study are available on request to the corresponding author.

## References

[1] Usmani Z., Sharma M., Awasthi A.K., Sharma G.D., Cysneiros D., Nayak S.C., Thakur V.K., Naidu R., Pandey A., Gupta V.K. (2021). Minimizing hazardous impact of food waste in a circular economy–Advances in resource recovery through green strategies. J. Hazard. Mater..

[2] Srivastava N., Srivastava M., Abd_Allah E.F., Singh R., Hashem A., Gupta V.K. (2021). Biohydrogen production using kitchen waste as the potential substrate: A sustainable approach. Chemosphere.

[3] European Commission (2020). A Farm to Fork Strategy for a Fair, Healthy and Environmentally Friendly Food System.

[4] Mena C., Adenso-Diaz B., Oznur Y. (2011). The causes of food waste in the supplier–retailer interface: Evidences from the UK and Spain. Resour. Conserv. Recycl..

[5] Green A., Johnston N., Waldron K., Faulds C., Smith A. (2004). Food surplus; reduction, recovery and recycle. Total Foods.

[6] Nellman C., MacDevette M., Manders T., Eickhout B., Svihus B., Prins A.G., Kaltenborn B.P. (2009). The Environmental Food Crisis–the Environment’s Role in Averting Future Food Crises.

[7] Forkes J. (2007). Nitrogen balance for the urban food metabolism of Toronto. Resour. Conserv. Recycl..

[8] Lundqvist J., de Fraiture C., Molden D. (2008). Saving water: From field to fork–curbing losses and wastage in the food chain. Stockh. Int. Water Inst. Stockholm.

[9] Griffin M., Sobal J., Lyson T.A. (2009). An analysis of a community food waste stream. Agric. Hum. Values.

[10] Hogg D., Barth J., Scheliss K., Favoino E. (2007). Dealing with Food Waste in the UK London.

[11] Henderson G. (2004). ‘Free’ food, the local production of worth, and the circuit of decommodification: A value theory of the surplus. Environ. Plan. D Soc. Space.

[12] Ventour L. (2008). The Food We Waste: Food Waste Report v2.

[13] Gaiani S., Caldeira S., Adorno V., Segrè A., Vittuari M. (2018). Food wasters: Profiling consumers’ attitude to waste food in Italy. Waste Manag..

[14] Bravia L., Francioni B., Murmuraa F., Savellia E. (2020). Factors affecting household food waste among young consumers and actions to prevent it. A comparison among UK, Spain and Italy. Resour. Conserv. Recycl..

[15] Mirabella N., Castellani V., Sala S. (2014). Current options for the valorization of food manufacturing waste: A review. J. Clean. Prod..

[16] Dreyer H.C., Dukovska-Popovska I., Yu Q., Hedenstierna C.P. (2019). A ranking method for prioritising retail store food waste based on monetary and environmental impacts. J. Clean. Prod..

[17] Parizeau K., von Massow M., Martin R. (2015). Household-level dynamics of food waste production and related beliefs, attitudes, and behaviours in Guelph, Ontario. Waste Manag..

[18] Muriana C. (2017). A focus on the state of the art of food waste/losses issue and suggestions for future researches. Waste Manag..

[19] Aschemann-Witzel J., Giménez A., Ares G. (2018). Convenience or price orientation? Consumer characteristics influencing food waste behaviour in the context of an emerging country and the impact on future sustainability of the global food sector. Glob. Environ. Chang..

[20] Romani S., Grappi S., Bagozzi R.P., Barone A.M. (2018). Domestic food practices: A study of food management behaviors and the role of food preparation planning in reducing waste. Appetite.

[21] European Commission (2016). Estimates of European Food Waste Levels. http://www.eu-fusions.org/phocadownload/Publications/Estimates%20of%20European%20food%20waste%20levels.pdf.

[22] Stenmarck Â., Jensen C., Quested T., Moates G., Buksti M., Cseh B., Scherhaufer S. (2016). Estimates of European Food Waste Levels.

[23] European Commission (2015). EU Actions against Food Waste. http://ec.europa.eu/food/safety/food_waste/eu_actions/index_en.htm/.

[24] Roodhuyzen D.M.A., Luning P.A., Fogliano V., Steenbekkers L.P.A. (2017). Putting together the puzzle of consumer food waste: Towards an integral perspective. Trends Food Sci. Technol..

[25] Read Q., Brown S., Cuellar A., Finn S., Gephart J., Marston L., Meyer E., Weitz K., Muth M. (2020). Assessing the environmental impacts of halving food loss and waste along the food supply chain. Sci. Total Environ..

[26] Skaf L., Franzese P.P., Capone R., Buonocore E. (2021). Unfolding hidden environmental impacts of food waste: An assessment for fifteen countries of the world. J. Clean. Prod..

[27] Di Talia E., Simeone M., Scarpato D. (2019). Consumer behaviour types in household food waste. J. Clean. Prod..

[28] Visschers V.H., Wickli N., Siegrist M. (2016). Sorting out food waste behaviour: A survey on the motivators and barriers of self-reported amounts of food waste in households. J. Environ. Psychol..

[29] Schanes K., Dobernig K., G€ozet B. (2018). Food waste matters—A systematic review of household food waste practices and their policy implications. J. Clean. Prod..

[30] Attiq S., Habib M.D., Kaur P., Hasni M.J.S., Dhir A. (2021). Drivers of food waste reduction behaviour in the household context. Food Qual. Prefer..

[31] Olavarria-Key N., Ding A., Legendre T.S., Min J. (2021). Communication of food waste messages: The effects of communication modality, presentation order, and mindfulness on food waste reduction intention. Int. J. Hosp. Manag..

[32] Read Q.D., Muth M.K. (2021). Cost-effectiveness of four food waste interventions: Is food waste reduction a “win–win?”. Resour. Conserv. Recycl..

[33] Chawla G., Lugosi P., Hawkins R. (2020). Evaluating materiality in food waste reduction interventions. Ann. Tour. Res..

[34] Lin B., Guan C. (2021). Determinants of household food waste reduction intention in China: The role of perceived government control. J. Environ. Manag..

[35] Thyberg K.L., Tonjes D.J. (2016). Drivers of food waste and their implications for sustainable policy development. Resour. Conserv. Recycl..

[36] Mena C., Whitehead P. (2008). Evidence on the Role of Supplier-Retailer Trading Relationships and Practices in Waste Generation in the Food Chain.

[37] Mena C., Terry L.A., Williams A., Ellram L. (2014). Causes of waste across multi-tier supply networks: Cases in the UK food sector. Int. J. Prod. Econ..

[38] Jeswani H.K., Figueroa-Torres G., Azapagic A. (2021). The extent of food waste generation in the UK and its environmental impacts. Sustain. Prod. Consum..

[39] Dumitru O.M., Iorga S.C., Sanmartin Á.M. (2021). Food waste impact on Romanian households. Rom. Biotechnol. Lett..

